# The major causes and risk factors of total and cause-specific mortality during 5.4-year follow-up: the Shanghai Changfeng Study

**DOI:** 10.1007/s10654-019-00543-0

**Published:** 2019-08-01

**Authors:** Li Wu, Huandong Lin, Yu Hu, Chouwen Zhu, Hui Ma, Jian Gao, Jiong Wu, Hong Shen, Wenhai Jiang, Naiqing Zhao, Yiqing Yin, Baishen Pan, Johannes Jeekel, Albert Hofman, Xin Gao

**Affiliations:** 1 Department of Endocrinology and Metabolism, Zhongshan Hospital, Fudan University, Shanghai, 200032 China; 2 Fudan Institute for Metabolic Diseases, Shanghai, 200032 China; 3 Department of Geriatrics, Zhongshan Hospital, Fudan University, Shanghai, 200032 China; 4 Department of Gastroenterology, Zhongshan Hospital, Fudan University, Shanghai, 200032 China; 5 Department of Clinical Nutrition, Zhongshan Hospital, Fudan University, Shanghai, 200032 China; 6 Department of Laboratory Medicine, Zhongshan Hospital, Fudan University, Shanghai, 200032 China; 7 Network Information Center, Zhongshan Hospital, Fudan University, Shanghai, 200032 China; 8 Department of Biostatistics, School of Public Health, Fudan University, Shanghai, 200032 China; 9 Department of Neuroscience, Erasmus Medical Center, Rotterdam, The Netherlands; 10 Department of Epidemiology, Harvard T.H. Chan School of Public Health, Boston, MA USA; 11 State Key Laboratory of Genetic Engineering, Human Phenome Institute and School of Life Sciences, Fudan University, Shanghai, China

**Keywords:** Causes of death, Risk factors, Middle-aged and elderly Chinese population

## Abstract

To investigate the major causes and predictive factors of death in a middle-aged and elderly Chinese population. A total of 6591 residents aged ≥ 45 years from Shanghai Changfeng community were followed up for an average of 5.4 years. The causes of death were coded according to the 10th Revision of International Classification of Diseases. The mortality rate was calculated by person-years of follow up and age-standardized according to the 2010 Chinese census data. Multivariable-adjusted Cox proportional hazards model was performed to investigate the predictors of all-cause and cause-specific mortality. During the total follow-up of 35,739 person-years, 370 deaths were documented (157 from malignant neoplasms, 70 from heart diseases, 68 from cerebrovascular diseases, 75 from other causes). The age-standardized all-cause mortality rate was 798.2 per 100,000 person-years (927.9 among men and 716.7 among women). Results from multivariable analyses showed that aging, diabetes, and osteoporosis at baseline were independent predictors of all-cause mortality, with hazard ratios (HR) of 1.11 (95% CI 1.10–1.13), 1.91 (1.51–2.42), and 1.71 (1.24–2.35), respectively. The population attributable risk percent of diabetes and osteoporosis was 19.7% and 11.7%, respectively. Cigarette smoking was associated with a higher risk of all-cause mortality in men (HR and 95%CI 1.44, 1.01–2.06). In women, diabetes and osteoporosis were related to a higher risk of cardiovascular mortality (3.27, 1.82–5.88 and 1.89, 1.04–3.46, respectively). While in men, osteoporosis was related to a higher risk of malignant neoplasms mortality (2.39, 1.07–5.33). Malignant neoplasms, heart diseases, and cerebrovascular diseases are the leading causes of death. Aging, smoking, underweight, diabetes, and osteoporosis are independent predictors of premature death among middle-aged and elderly Chinese community population. Moreover, there may have been some differences in the causes and predictors of premature death between men and women.

## Introduction

Globally, rapid economic development and improvement in living conditions, nutrition, and health care have resulted in declines in infant mortality and deaths from infectious diseases and increases in life expectancy during the past three decades [1, 2]. In contrast, urbanized lifestyle (calorie-dense diet and sedentary environment) accompanied with the aging population have led to the rapid rise in prevalence of chronic non-communicable diseases (NCDs), such as cardiovascular diseases, cancers, chronic pulmonary diseases, and diabetes mellitus [3]. The Global Burden of Diseases Study 2016 (GBD 2016) reported that mortality due to NCDs increased from 57% of total mortality in 1990 to 72.3% in 2016 [1]. In China, with remarkable economic and social development, the life expectancy at birth increased by 8.5 years from 1990 to 2013 [4], and NCDs caused mortality has increased from 69.2 to 86.6% of the total annual deaths during the same period [5].

Changes in mortality and its main causes are important indicators of national health. China has been undergoing tremendous demographic and epidemiological transitions during the past three decades, but the leading health problems differ across different regions [5]. Local health policies are needed to be made to deal with the diverse challenges faced by local health-care systems. With the improvement of the death surveillance system, investigation of death causes and risk factors have become possible [6].

However, most previous death investigations were conducted among the whole population, the surveys focused on middle-aged and elderly population were limited. Gu et al. [7] ever estimated the major causes and preventable risk factors of mortality among Chinese adults aged 40 years or older from 1991 to 2000. Since then, there were few studies estimating the causes and risk factors for mortality among Chinese middle-aged and elderly population, and most of the existing ones were conducted in a specific population, such as the retired military veterans [8] or male steelworkers [9]. Shanghai is one of the most developed cities in China, the aging tendency of its population is more severe than most other Chinese cities. Valid information on mortality and its modifiable risk factors is crucial for health policy to set priority for the prevention of premature death. In this study, using data from the Shanghai Changfeng study [10, 11], we aimed to investigate the major causes and predictive factors of premature death in a Chinese community population aged 45 years and older.

## Methods

### Study population

The participants were from Shanghai Changfeng Study, a prospectively observational community-based cohort study, which is focused on chronic diseases among the middle-aged and elderly. Details of the study information have been described elsewhere [10]. The Changfeng community is a middle-class community located in the north-west part of Shanghai. The inclusion criteria of participants were: (1) aged 45 years or older, (2) lived in Changfeng community for at least 5 years. Our study population can represent the general adult population aged 45 years or older in Shanghai. From June 2009 to December 2012, 6595 residents were enrolled. During the follow-up period, 4 participants missing the vital status record were excluded. Eventually, 6591 individuals were included in our final analyses. This study was approved by the ethical committee of Zhongshan Hospital affiliated to Fudan University and informed consent was obtained from all individual participants.

### Baseline examination

Information on sociodemographic variables, including age, gender, cigarette smoking, and alcohol consumption, were collected through face-to-face interviews using structured questionnaires. The participants were instructed to maintain their usual lifestyle for at least 3 days before each examination. Cigarette smoker was defined as smoking at least one cigarette per day for one or more years. Regular alcohol consumption was defined as the average alcohol consumption of more than 10 g of absolute alcohol per day for 1 year or longer.

Height and weight were measured with the participants wearing light clothes without shoes. Body mass index (BMI) was calculated as weight in kilograms divided by the square of height in meters (kg/m^2^). Normal weight was defined as a BMI of 18.5 to < 24.0 kg/m^2^, underweight was defined as a BMI of < 18.5 kg/m^2^, overweight was defined as a BMI of 24.0 to < 28.0 kg/m^2^, and obesity as a BMI of ≥ 28.0 kg/m^2^ according to Chinese criteria [12]. Resting blood pressure was measured three times in the same arm with an electronic blood pressure monitor (OMRON Model HEM-752 FUZZY, Omron Co., Dalian, China) after the participants had been sitting quietly for at least 5 min, and the average of three measurements was used for analysis. Hypertension was defined as a blood pressure ≥ 140/90 mmHg or self-reported current use of antihypertensive medications, and pre-hypertension was defined as 120 ≤ systolic blood pressure (SBP) ≤ 139 mmHg or 80 ≤ diastolic blood pressure (DBP) ≤ 89 mmHg [13].

Blood samples were collected after a fasting period of at least 10 h. Fasting plasma glucose (FPG), total cholesterol (TC), triglyceride (TG), high-density lipoprotein cholesterol (HDL-C), and low-density lipoprotein cholesterol (LDL-C) were measured using an automated bio-analyzer (HITACHI 7600, Tokyo, Japan). Dyslipidemia was defined as TC ≥ 6.22 mmol/L, or TG ≥ 2.26 mmol/L, or LDL-C ≥ 4.14 mmol/L, or HDL-C < 1.04 mmol/L, or self-reported current use of antilipidemic agents [14]. The 2-h plasma glucose (2-h PG) was measured after a 75 g oral glucose tolerance test (OGTT). Diabetes mellitus (DM) was defined as FPG ≥ 7.0 mmol/L or 2-hPG ≥ 11.1 mmol/L according to the WHO 1999 criteria [15], or a previous diagnosis or self-reported current treatment with hypoglycemic drugs. Pre-diabetes was defined as either impaired fasting glucose (FPG ≥ 6.1 and < 7.0 mmol/L) or impaired glucose tolerance (2-h PG ≥ 7.8 and < 11.1 mmol/L). All blood samples were measured in the central laboratory of the Zhongshan Hospital affiliated to Fudan University.

Bone mineral density (BMD) at the lumbar spine (L1–L4), femoral neck, and total hip were measured using dual-energy X-ray absorptiometry (GE Healthcare Lunar iDXA system) and conducted by a trained technician. Osteopenia was defined as a T score of − 1.0 to − 2.5 standard deviations (SD) and osteoporosis as a T score ≤ −2.5 SD according to the WHO criteria [16].

### Follow-up

We followed participants from the date of recruitment to the date of death or 31 December 2016, whichever came first. The mean follow-up time was 5.4 years. The vital status of participants was collected from the Shanghai Centers for Disease Control (SCDC), and the causes of death were coded according to the 10th Revision of International Classification of Diseases (ICD-10). The main endpoints during the follow-up were death due to malignant neoplasms (ICD-10 codes C00-C97), heart diseases (ICD-10 codes I10-I52), cerebrovascular diseases (ICD-10 codes I60-I69), diabetes mellitus (ICD-10 codes E10-E14), respiratory diseases (ICD-10 codes J00-J99), digestive diseases (ICD-10 codes K00-K93), dementia (ICD-10 codes F00-F03) and accidents (ICD-10 codes V01-X59). All-cause mortality was defined as death due to any causes. Cardiovascular mortality was defined as death caused by coronary disease, stroke, and peripheral arterial disease. Malignant neoplasms mortality was defined as death due to malignant neoplasms.

### Statistical analysis

Continuous variables were presented as mean ± standard deviation (SD) and categorical variables were presented as numbers (percentages). *T* test was used for comparison of continuous variables, whereas the Chi squared test was used for comparisons of categorical variables. The baseline characteristics of participants were compared between men and women.

Participants contributed follow-up time was calculated from the date of recruitment to the date of death or 31 December 2016, whichever came first. Age-standardized mortality was calculated by person-years of follow up and adjusted according to the 2010 census data in China [17]. Multivariable-adjusted Cox proportional hazards model was performed to estimate the hazard ratio (HR) and 95% confidence interval (95% CI) of predictive factors for all-cause and cause-specific mortality. Potential confounders included baseline age, gender, alcohol consumption, cigarette smoking, and existing diseases (yes/no) including obesity, diabetes, hypertension, dyslipidemia, and osteoporosis. To further analyze the associations of baseline age, BMI, glucose metabolism, blood pressure, bone mineral density with all-cause mortality, we introduced dummy variables in Cox analyses. Participants were divided into different groups according to age [45–54 (reference), 55–64, 65–74, and ≥ 75 years old], BMI [< 18.5, 18.5 to < 24.0 (reference), 24.0 to < 28.0, and ≥ 28.0 kg/m^2^], glucose metabolism [Normal (reference), Pre-DM, New DM, and Prior DM], blood pressure [Normal (reference), Pre-hypertension, New hypertension, and Prior hypertension] and bone mineral density [Normal (reference), Osteopenia, and Osteoporosis], respectively. The HR of all-cause mortality in one group as compared with the reference group was calculated using multivariable-adjusted Cox proportional hazards model. When one of these factors was categorized and analyzed, the other four factors were adjusted, as well as gender, cigarette smoking, alcohol drinking, and dyslipidemia. Stratified analyses according to sex were performed in all multivariable-adjusted Cox analyses. Population attributable risk percent (PAR%) was calculated following the formula: PAR% = P_e_ (RR − 1)/[1 + P_e_ (RR − 1)] [18], where P_e_ represented the proportion of exposure in the total population, and HR was considered as RR in Cox analysis. All analyses were performed using SPSS version 19.0. Statistical tests were two-sided and a *P *< 0.05 was considered statistically significant.

## Results

### Baseline characteristics and major causes of death in the study population

As shown in Table 1, the mean age of participants was 63.7 years (46.0–95.8 years) at baseline, and 42.5% of them were men. Compared to women, men were more likely to be cigarette smokers or alcohol consumers, to have hypertension, dyslipidemia or diabetes, but less likely to have osteoporosis (all *P *< 0.001).

**Table 1:** Baseline characteristics of participants

	Total (N = 6591)	Men (N = 2804)	Women (N = 3787)	*P*
Age (years)	63.7 ± 9.7	64.5 ± 9.8	63.0 ± 9,6	< 0.001
BMI (kg/m^2^)	24.3 ± 3.3	24.5 ± 3.1	24.1 ± 3.5	< 0.001
WC (cm)	84.0 ± 9.7	87.0 ± 9.2	81.9 ± 9.5	< 0.001
SBP (mmHg)	135.8 ± 19.2	137.2 ± 18.3	134.7 ± 20.0	< 0.001
DBP (mmHg)	76.3 ± 10.2	78.4 ± 10.3	74.8 ± 9.8	< 0.001
TC (mmol/L)	5.07 ± 0.94	4.76 ± 0.88	5.29 ± 0.92	< 0.001
TG (mmol/L)	1.71 ± 1.23	1.72 ± 1.26	1.70 ± 1.20	0.601
HDL-C (mmol/L)	1.43 ± 0.38	1.29 ± 0.32	1.53 ± 0.38	< 0.001
LDL-C (mmol/L)	2.89 ± 0.81	2.72 ± 0.76	3.01 ± 0.81	< 0.001
FPG (mmol/L)	5.63 ± 1.55	5.75 ± 1.70	5.54 ± 1.43	< 0.001
2-h PG (mmol/L)	7.63 ± 3.31	7.86 ± 3.45	7.47 ± 3.19	< 0.001
Smokers, n (%)	1496 (22.7)	1415 (50.6)	81 (2.1)	< 0.001
Regular alcohol consumers, n (%)	1089 (16.6)	948 (33.9)	141 (3.7)	< 0.001
Existing diseases				
Obesity, n (%)	806 (12.4)	327 (11.8)	479 (12.8)	0.241
Diabetes, n (%)	1448 (22.3)	703 (25.4)	745 (19.9)	< 0.001
Hypertension, n (%)	3636 (55.6)	1674 (60.2)	1962 (52.2)	< 0.001
Dyslipidemia, n (%)	2172 (33.1)	979 (35.1)	1193 (31.6)	0.003
Osteoporosis, n (%)	576 (9.4)	71 (2.7)	505 (14.3)	< 0.001

Data are presented as mean ± standard deviations or as number (percentage)

*BMI* body mass index, *WC* waist circumference, *SBP* systolic blood pressure, *DBP* diastolic blood pressure, *TC* total cholesterol, *TG* triglyceride, *LDL-C* low-density lipoprotein cholesterol, *HDL-C* high-density lipoprotein cholesterol, *FPG* fasting plasma glucose, *2-h PG* 2-hour plasma glucose in 75-g oral glucose tolerance test

**Fig. 1:**
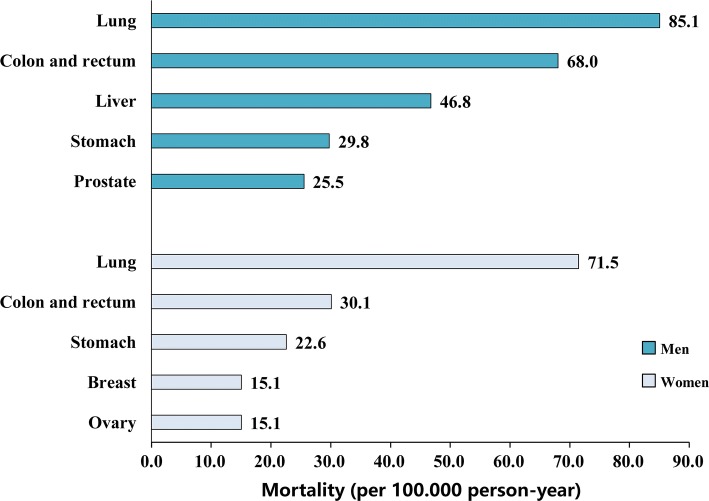
Age-standardized mortality for the five leading causes of death from malignant neoplasms

During the mean follow-up of 5.4 years (35,739 person-years in total), we have documented 370 deaths, including 157 from malignant neoplasms, 70 from heart diseases, 68 from cerebrovascular diseases, 26 from diabetes, 11 from respiratory diseases, 10 from digestive diseases, 8 from accidents, 3 from dementia, and the rest from other causes. The age-standardized mortality rate was 798.2 per 100,000 person-years (927.9 per 100,000 person-years among men and 716.7 per 100,000 person-years among women). Age-standardized mortality rate and the percentage of total deaths for the eight leading causes of death are shown in Table 2. Overall, the three leading causes of death (malignant neoplasms, heart diseases, and cerebrovascular diseases) accounted for 79.7% of total deaths (76.2% among men and 84.3% among women). The distribution of most cause-specific mortality was similar between men and women, except for mortality from respiratory diseases and accidents, which were higher in men than women. Moreover, the five leading causes of death from malignant neoplasms were cancer of the lung, colon and rectum, liver, stomach, and prostate in men, and cancer of the lung, colon and rectum, stomach, breast, and ovary in women (Fig. 1).

**Table 2:** Age-standardized mortality and the percentage of total deaths for the eight leading causes of death in the middle-aged and elderly Chinese population

Cause of death^a^	No. of deaths	Mortality (per 100,000 person-year)	Age-standardized mortality (per 100,000 person-year)^b^	Percentage of total deaths	Rank order
All causes					
Total	370	1035.3	798.2	100.0	–
Men	210	1399.0	927.9	100.0	–
Women	160	773.0	716.7	100.0	–
Malignant neoplasms					
Total	157	439.3	312.1	42.4	1
Men	87	579.6	370.0	41.4	1
Women	70	338.2	263.4	43.8	1
Heart diseases^c^					
Total	70	195.9	170.7	18.9	2
Men	36	239.8	161.6	17.1	3
Women	34	164.3	190.5	21.3	2
Cerebrovascular diseases					
Total	68	190.3	160.2	18.4	3
Men	37	246.5	184.5	17.6	2
Women	31	149.8	154.2	19.4	3
Diabetes mellitus					
Total	26	72.8	51.6	7.0	4
Men	15	99.9	63.8	7.1	4
Women	11	53.1	36.5	6.9	4
Respiratory diseases					
Total	11	30.8	22.9	3.0	5
Men	10	66.6	39.7	4.8	5
Women	1	4.8	2.6	0.6	8
Digestive diseases					
Total	10	28.0	21.2	2.7	6
Men	6	40.0	26.6	2.9	6
Women	4	19.3	15.9	2.5	5
Accidents^d^					
Total	8	22.4	13.4	2.2	7
Men	6	40.0	21.6	2.9	7
Women	2	9.7	5.1	1.3	6
Dementia					
Total	3	8.4	5.4	0.8	8
Men	1	6.7	2.8	0.5	8
Women	2	9.7	8.2	1.3	7
All other causes					
Total	17	47.6	40.7	4.6	–
Men	12	79.9	57.3	5.7	–
Women	5	24.2	40.3	3.1	–

^a^Causes of death were coded according to the *International Classification of Diseases, Tenth Revision* (ICD-10). Percentages do not necessarily sum to 100 because of rounding

^b^Age-standardized mortality (per 100,000 person-years) was calculated according to the 2010 census data for China’s population

^c^Heart diseases included acute rheumatic fever, chronic rheumatic heart disease, hypertensive disease, ischemic heart disease, diseases of the pulmonary circulation, and other forms of heart disease

^d^Accidents included vehicular accidents, falls, drowning, and poisoning

### Predictors of all-cause mortality

The multivariable-adjusted HR and PAR% of all-cause mortality for major predictive factors are shown in Table 3. Aging was the independent predictor of all-cause mortality, with an HR of 1.11 (95%CI1.10–1.13) per 1-year increment. Diabetes was responsible for 19.7% of the total mortality, with an HR of 1.91 (95%CI 1.51–2.42). In participants with diabetes, the risk of all-cause mortality was higher among women than men (HR 2.34 vs. 1.61). Osteoporosis accounted for 11.7% of the total mortality, with an HR of 1.71 (95%CI 1.24–2.35). Cigarette smoking significantly increased the risk of all-cause mortality among men, with an HR of 1.44 (95%CI 1.01–2.06) and PAR% of 53.5%. However, the presence of hypertension, obesity or dyslipidemia at baseline was not associated with the increased risk of all-cause mortality in our study.

**Table 3:** Hazard ratio and population attributable risk percent of predictors for all-cause mortality in multivariable-adjusted^a^ analyses

	Total		Men		Women	
	HR (95%CI)	PAR%	HR (95%CI)	PAR%	HR (95%CI)	PAR%
Age (years)	1.11 (1.10–1.13)	–	1.11 (1.09–1.13)	–	1.11 (1.09–1.13)	–
Smoking	1.29 (0.93–1.80)	–	1.44 (1.01–2.06)	53.5	0.78 (0.24–2.57)	–
Drinking	1.01 (0.72–1.42)	–	0.86 (0.59–1.26)	–	2.02 (0.96–4.25)	–
Obesity	1.18 (0.86–1.63)	–	1.27 (0.82–1.99)	–	1.10 (0.69–1.73)	–
Diabetes	1.91 (1.51–2.42)	19.7	1.61 (1.18–2.20)	29.5	2.34 (1.62–3.37)	12.9
Hypertension	1.06 (0.81–1.40)	–	1.05 (0.74–1.51)	–	1.09 (0.71–1.68)	–
Dyslipidemia	1.08 (0.85–1.37)	–	0.97 (0.70–1.36)	–	1.23 (0.87–1.75)	–
Osteoporosis	1.71 (1.24–2.35)	11.7	2.51 (1.50–4.21)	1.8	1.48 (1.00–2.21)	22.8

*HR* hazard ratio, *95%CI* 95% confidence interval, *PAR%* population attributable risk percent

^a^Adjusted for age, gender, cigarette smoking, alcohol drinking, and existing diseases (obesity, diabetes, hypertension, dyslipidemia, and osteoporosis) at baseline

As shown in Table 4, the HR of all-cause mortality in participants aged ≥ 75 years was nearly 26.6 times of that in participants aged 45–54 years. Participants who were underweight had a significantly higher risk of all-cause mortality than participants with normal weight (HR, 95%CI 1.73, 1.04–2.88). Osteoporosis independently predicted the risk of all-cause mortality among men, but not among women. Osteopenia could not predict the risk of all-cause mortality either among men or women. However, hypertension still had no significant association with all-cause mortality even we categorized it into pre-hypertension, newly diagnosed hypertension, and prior known hypertension.

**Table 4:** Adjusted^a^ hazard ratio and 95% confidence interval of all-cause mortality by various characteristics of participants

	Total		Men		Women	
	HR	95%CI	HR	95%CI	HR	95%CI
Age (years)						
45–54^Ref.^	1.00	Ref.	1.00	Ref.	1.00	Ref.
55–64	3.72	1.68–8.26	3.08	1.06–8.96	4.95	1.38–15.32
65–74	9.84	4.52–21.43	11.13	4.00–31.01	8.03	2.41–26.76
≥ 75	26.57	12.27–57.54	25.61	9.22–71.11	27.00	8.24–88.45
BMI (kg/m^2^)						
< 18.5	1.73	1.04–2.88	2.04	1.08–3.86	1.46	0.62–3.44
18.5 to < 24.0^Ref.^	1.00	Ref.	1.00	Ref.	1.00	Ref.
24.0 to < 28.0	0.81	0.62–1.05	0.71	0.50–1.01	0.98	0.65–1.48
≥ 28.0	1.07	0.76–1.52	1.09	0.68–1.76	1.09	0.65–1.82
Glucose metabolism						
Normal^Ref.^	1.00	Ref.	1.00	Ref.	1.00	Ref.
Pre-DM	1.11	0.81–1.51	1.11	0.74–1.66	1.11	0.68–1.81
New DM	1.80	1.30–2.49	1.34	0.86–2.09	2.46	1.51–4.03
Prior DM	2.17	1.60–2.96	2.02	1.35–3.01	2.42	1.47–3.97
Blood pressure (mmHg)						
Normal^Ref.^	1.00	Ref.	1.00	Ref.	1.00	Ref.
Pre-hypertension	0.90	0.54–1.48	0.63	0.33–1.20	1.43	0.64–3.21
New hypertension	0.59	0.34–1.03	0.46	0.23–0.93	0.84	0.34–2.11
Prior hypertension	1.11	0.70–1.77	0.85	0.47–1.53	1.60	0.75–3.42
Bone mass						
Normal^Ref.^	1.00	Ref.	1.00	Ref.	1.00	Ref.
Osteopenia	1.25	0.96–1.64	1.22	0.88–1.70	1.18	0.73–1.90
Osteoporosis	1.98	1.37–2.86	2.72	1.59–4.64	1.69	0.98–2.90

*HR* hazard ratio, *95%CI* 95% confidence interval, *BMI* body mass index, *DM* diabetes mellitus

^a^Multivariable-adjusted Cox regression analyses, when one of these factors was categorized and analyzed, the other four factors were adjusted, as well as gender, cigarette smoking, alcohol drinking, and dyslipidemia

^Ref.^The reference group was the 45–54 years age group, the 18.5 to < 24.0 kg/m^2^ group, the normal glucose metabolism group, the normal blood pressure group, and the normal bone mineral density group, respectively

### Predictors of cardiovascular diseases and malignant neoplasms mortality

As shown in Table 5, the risk of cardiovascular mortality increased by 13.6% (12.8% among men and 15.0% among women) with per 1-year increase in age. Diabetes and osteoporosis independently predicted the risk of cardiovascular mortality among women, with an HR of 3.27 (95%CI 1.82–5.88) and 1.89 (95%CI 1.04–3.46) respectively, but not among men. The risk of malignant neoplasms mortality increased by 9.9% (9.8% among men and 9.6% among women) with per 1-year increase in age. Compared with participants with normal glucose regulation, the risk of malignant neoplasms mortality increased by 49% in patients with diabetes (HR, 95%CI 1.49, 1.03–2.14). Osteoporosis independently predicted the risk of malignant neoplasms mortality among men, with an HR of 2.39 (95%CI 1.07–5.33), but not among women.

**Table 5:** Adjusted^a^ hazard ratio and 95% confidence interval of predictors for cardiovascular and malignant neoplasms mortality

	Total		Men		Women	
	HR	95%CI	HR	95%CI	HR	95%CI
Cardiovascular mortality						
Age (years)	1.14	(1.11–1.16)	1.13	(1.09–1.17)	1.15	(1.11–1.20)
Smoking	1.57	(0.90–2.76)	1.77	(0.96–3.26)	–	–
Drinking	0.88	(0.48–1.60)	0.84	(0.44–1.61)	0.81	(0.11–5.90)
Obesity	0.98	(0.56–1.72)	1.36	(0.65–2.85)	0.65	(0.27–1.57)
Diabetes	2.03	(1.37–3.00)	1.36	(0.79–2.34)	3.27	(1.82–5.88)
Hypertension	1.52	(0.91–2.54)	1.86	(0.89–3.88)	1.17	(0.56–2.42)
Dyslipidemia	1.212	(0.82–1.80)	1.26	(0.73–2.20)	1.10	(0.62–1.95)
Osteoporosis	1.92	(1.16–3.18)	2.31	(0.90–5.95)	1.89	(1.04–3.46)
Malignant neoplasms mortality						
Age (years)	1.10	(1.08–1.12)	1.10	(1.07–1.13)	1.10	(1.06–1.13)
Smoking	1.04	(0.63–1.73)	1.15	(0.67–1.99)	1.12	(0.26–4.93)
Drinking	1.12	(0.67–1.87)	0.90	(0.51–1.59)	2.56	(0.98–6.67)
Obesity	1.25	(0.78–1.99)	0.81	(0.37–1.80)	1.72	(0.93–3.16)
Diabetes	1.49	(1.03–2.14)	1.50	(0.93–2.42)	1.39	(0.79–2.45)
Hypertension	0.83	(0.56–1.22)	0.83	(0.50–1.39)	0.86	(0.47–1.55)
Dyslipidemia	1.23	(0.87–1.75)	1.10	(0.67–1.81)	1.56	(0.93–2.62)
Osteoporosis	1.21	(0.72–2.03)	2.39	(1.07–5.33)	0.93	(0.48–1.79)

*HR* hazard ratio, *95%CI* 95% confidence interval

^a^Adjusted for age, gender, cigarette smoking, alcohol drinking, and existing diseases (obesity, diabetes, hypertension, dyslipidemia, and osteoporosis) at baseline

## Discussion

In the present study, we found that malignant neoplasms, heart diseases, and cerebrovascular diseases were the three leading causes of death and together accounted for approximately four-fifths of the total mortality. After them, diabetes mellitus was the fourth leading cause of death. Aging, smoking, underweight, diabetes, and osteoporosis were the independent predictors of death.

Monitoring the levels and trends in mortality is crucial to determining how societies can address prominent sources for premature death. Our findings provide important implications for public health. In a recent global study [19], Foreman and colleagues developed a new model and used data from the GBD 2016 study [1] to forecast life expectancy, all-cause mortality, and risk factors of death from 2016 to 2040. Their results showed that NCDs would account for a higher proportion of years of life lost (YLLs) by 2040 (67·3% of YLLs [95% UI 61·9–72·3] globally) than 2016. YLLs were calculated from the sum of each death multiplied by the standard life expectancy at each age. For most countries, prioritizing NCDs and NCD-related risks in health planning and investment decisions has the potential to markedly reduce premature mortality by 2040. In our study, NCDs (malignant neoplasms, heart diseases, cerebrovascular diseases, and diabetes) were the leading causes of death. These results were mostly consistent with Foreman’s study. However, diabetes has become the fourth leading cause of death in our population but ranked as the eighth leading cause in Foreman’s study. This observation may be due to the substantial increases in the prevalence of diabetes during the past three decades in China, especially among the elderly population. We also found that the distribution of most cause-specific mortality was similar between men and women, except for respiratory diseases and accidents, this may because men are more likely to be cigarette smokers and have traffic accidents than women.

The malignant neoplasm was the first leading cause of death in our study, the five leading causes of death from malignant neoplasms were lung, colon and rectum, liver, stomach, and prostate cancer among men, and lung, colon and rectum, stomach, breast, and ovarian cancer among women. These results were substantially consistent with the Chinese cancer mortality report in 2018 [20]. However, the ratio of mortality caused by malignant neoplasms in our study was higher than that report; this may be because all of our participants were older than 45 years [21]. In China, lung cancer, female breast cancer, stomach cancer, liver cancer, esophageal cancer, as well as colorectal cancer have become the major causes of malignant neoplasm mortality and resulted in severe burden to our society. Efficient interventions and control strategies are urgently needed, such as changing from calorie-dense diet and sedentary lifestyle to fresh fruits and vegetables-rich diet and physically active lifestyle, improving national cancer screening programs for common cancers, and so on.

Diabetes is a well-recognized risk factor for cardiovascular and cerebrovascular diseases worldwide [22–24]. Convincing evidence indicates that diabetes is also associated with increased risk of several malignant neoplasms [25]. Coughlin et al. [26] and Gross et al. [27] found that diabetes was associated with a higher risk of death from colon, liver, and pancreatic cancer. However, the causal relationship between diabetes and malignant neoplasms has not been established, further researches are needed. Since 1980, the prevalence of diabetes has been quadrupled worldwide [28]. In China, the prevalence of diabetes has increased from 0.67% in 1980 to 11.6% in 2010, whereas rates of awareness, treatment, and control remain unacceptably low [29–32]. Our results showed that diabetes was the leading predictive factor of all-cause mortality, cardiovascular mortality, and malignant neoplasms mortality, and diabetes was responsible for 19.7% of the all-cause mortality. These results were consistent with previous studies which suggested that diabetes was closely associated with death from all causes, cardiovascular diseases, and malignant neoplasms [33–35]. Coughlin et al. [26] and Gross et al. [27] found that diabetes was associated with a higher risk of death from colon, liver, and pancreatic cancer. Even so, the causal relationship between diabetes and malignant neoplasms has not been established, further researches are needed.

Except for traditional risk factors such as aging, cigarette smoking, underweight, and diabetes, we also found that osteoporosis was an independent predictor of all-cause and malignant neoplasms caused mortality among men, and cardiovascular mortality among women, this observation was not mentioned in the Foreman’s study [19]. The association between osteoporosis and death may be mediated by osteoporotic fracture, which has become a major cause of death in the elderly population [36]. Osteoporosis is common in older women because of progressive postmenopausal bone loss due to the decreasing of estrogen levels. Although the prevalence of osteoporosis in men was significantly lower than women whether in our study or other existing data [37], men with osteoporosis might have higher mortality than women [38]. The causes of osteoporosis in men were more complex than that in women, such as androgen deprivation therapy for prostate cancer and alcohol excess. These may partly explain why osteoporosis can predict the risk of malignant neoplasms mortality among men. In line with our results, previous studies also found that osteoporosis was closely associated with the increased risk of cardiovascular diseases and mortality, especially among women [39, 40]. Further studies are needed to elucidate the mechanism of these associations.

In our study, cigarette smokers had a 44% higher risk of all-cause mortality than those who never smoked among men, but not among women. This is mainly because of the limited statistical power, because much fewer women (2.1%) smoked than men (50.6%) in our population. Our results were consistent with those from several prospective cohort studies and case–control studies conducted in China [41–43]. Underweight was also an independent predictor of all-cause mortality in our population. The risk of all-cause mortality increased by 73% in participants who were underweight than those with normal weight. Underweight reflected malnutrition and was considered as the leading cause of the disease burden in China 15 years ago [44], but now it is mainly caused by unhealthy diets, malignant neoplasms, and other consumptive diseases, all of which can increase the risk of death.

However, hypertension, obesity, and dyslipidemia were not related with the risk of all-cause mortality in our study population; even if we categorized our participants into pre-hypertension, newly diagnosed hypertension, and previously diagnosed hypertension. We think these may be because most patients with hypertension or dyslipidemia began to take a medication once they were diagnosed, and their blood pressure and serum lipid were controlled within the normal range in most time. One other explanation might be that the follow-up time in our study was not long enough to see the impact of hypertension and dyslipidemia on mortality. The association between obesity and all-cause mortality is still controversial [45, 46]. This may due to the diagnostic criterion for obesity is different across studies, and most of our obesity participants had a BMI of 28.0 to < 35 kg/m^2^, which was considered as mild obesity. Flegal et al. [47] reported that mild obesity (BMI 30 to < 35 kg/m^2^) was not associated with higher all-cause mortality, their results were consistent with ours.

In the present study, using a large prospective cohort with long-term follow-up, we identified the major causes of death and its predictors in a representative sample of the middle-aged and elderly population, providing some guidance for government to make health policies. Nevertheless, a few limitations need to be noticed in our study. Firstly, we did not have autopsy data to confirm the causes of death; this may induce some misclassification bias. However, this is the most practical way in epidemiology studies. Secondly, factors such as education, marital status, social economic status, diet quality, and physical activity were not included in our analyses partially because of missing values, and this will lead to residual confounding. Thirdly, as the death toll was relatively small based on the current follow-up time in our study, the impact of some risk factors on mortality may not be yet apparent, such as hypertension and dyslipidemia.

In conclusion, our study indicated that malignant neoplasms, heart diseases, and cerebrovascular diseases were three leading causes of death in middle-aged and elderly Chinese community population. Moreover, malignant neoplasm has become the first leading cause of death instead of heart diseases, and diabetes has risen to the fourth cause of death. Aging, smoking, underweight, diabetes, and osteoporosis were the independent predictors for premature death, and their effect on death might be different between men and women. Efficient interventions and control strategies are urgently needed, such as changing from calorie-dense diet and sedentary lifestyle to diets rich in fresh fruits and vegetables as well as a physically active lifestyle, and promoting national cancer screening programs for common cancers. Beyond that, prevention and control of diabetes and osteoporosis, quit smoking, and maintain a healthy weight are also very important for reducing the burden of premature mortality.
